# The burden of pharmacological treatment on health-related quality of life in people with a urea cycle disorder: a qualitative study

**DOI:** 10.1186/s41687-021-00387-x

**Published:** 2021-10-25

**Authors:** Gillian Yeowell, Danielle Stephanie Burns, Francis Fatoye

**Affiliations:** Department of Health Professions, Manchester Metropolitan University, 53 Bonsall Street, Manchester, M15 6GX UK

**Keywords:** Urea cycle disorders, Sodium phenylbutyrate, Sodium benzoate, Glycerol phenylbutyrate, Quality of life, Pharmacological burden, Social, Psychological health, Physical health

## Abstract

**Background:**

Urea cycle disorders (UCD) are inborn errors of metabolism, typically presenting neonatally. Excess ammonia builds rapidly within the body risking hyperammonemic episodes and potentially death. Long-term management of the condition includes restrictive protein consumption, pharmacological interventions and, in extreme cases, liver transplantation. Pharmacological treatments such as sodium benzoate and sodium phenylbutyrate have proven effective but not without a multitude of negative attributes including poor taste, higher dosage and associated gastrointestinal discomfort that impacts health-related quality of life. Glycerol phenylbutyrate (GPB) has recently become a widely available pharmacological treatment with early reports of improved qualities, including taste and administration method. The following study aims to explore the burden of pharmacological treatment and the effects of the transition to GPB on health-related quality of life in people with a UCD.

**Results:**

Nine carers of children living with a UCD (mean age = 12.44, SD = 10.26) were interviewed regarding their experiences of pharmacological treatment in relation to their, and their child’s, health-related quality of life after transitioning to GPB. Three main themes were identified: psychological health, physical health and social participation. Carers struggled with anxiety surrounding their child’s condition and the battle of administering medication. Medication administration was perceived to have improved since the transition to GPB, alleviating distress for both carer and child. Issues involving school were described, ranging from difficulties integrating their child into mainstream schooling and the impact of treatment on participation in school and extracurricular activities. Carers encountered issues sourcing syringes to administer GPB, which induced stress. It could be suggested that some burden had been relieved by the transition to GPB. However, it appeared that difficulties associated with the illness would persist despite treatment, owing to the continuing nature of the condition.

**Conclusions:**

Adhering to a strict pharmacological regime caused immense stress for both carers and children, severely impacting on typical social activities such as eating at a restaurant or going on holiday. GPB was perceived to have alleviated some burden in terms of administration given improved characteristics concerning taste and dosage, important characteristics for both carers and children living with UCD. Practitioners should consider these findings when making clinical decisions for children with UCD and the effect of pharmacological treatment on carer’s health-related quality of life. Outreach work to facilitate greater understanding of the condition should be conducted with key locations, such as children’s schools. This would also help to alleviate carer burden.

## Background

Urea cycle disorders (UCDs) are rare metabolic disorders caused by inherited deficiencies in any one of the six enzymes or two transporters of the urea cycle pathway. It is estimated that UCD affects approximately 1:35,000 births although this figure could be higher when considering partial defects [1]. The urea cycle normally converts ammonia into urea for excretion. However, in people with a UCD, this does not happen and as a consequence, they face rapidly increasing ammonia levels that can be devastating to the body. Excess ammonia can cause significant brain damage resembling hypoxic-ischemic events, influencing child development [1]. Over half of clinicians would target > 50 μmol/L as a manageable ammonia level for UCD patients [2], where a normal reference range is typically ≤ 100 μmol/L for neonates and ≤ 50 μmol/L beyond the neonatal period [3].

Diagnosing a UCD early is critical to reducing mortality and potential permanent neurological damage [4]. Initial symptoms of the disease include lethargy, non-compliance to diet and abnormal motor function, which typically precedes the first hyperammonemic episode [5]. Non-compliance to diet can manifest itself as protein aversion, poor appetite and adverse reactions to high-protein-containing foods [6]. Whilst these symptoms often present in the neonatal period, there are cases of late onset UCD in adult patients that have been triggered by a significant life event such as pregnancy [7]. Survival of UCD patients drops dramatically during the first 5 years of life after neonatal onset [8]. Earlier studies show that the mortality rate in neonatal onset UCD cases was 24% and 11% in late onset cases [9]. However, ongoing research suggests life expectancy has increased within the last 10 years due to the introduction of new screening and treatments [10]. As a consequence, there is an ever-increasing number of families who engage in long-term management of the condition owing to a growing number of treatment options [11].

UCDs are normally treated in a multifaceted way dependent on the severity of the disease. These methods include: strict dietary control involving heavily restricted protein intake, pharmacological interventions, and amino acid intake [12]. Pharmaceutical interventions are frequently used to control ammonia levels. Two of the most commonly prescribed drugs, sodium phenylbutyrate (NaPBA) and sodium benzoate (NaBZ), remove excess ammonia from the body. Each treatment can be taken in a variety of forms including granules, tablets and liquid-based. Patients who have taken NaPBA orally have generally reported negative attributes such as: poor tolerability, extremely high burden of treatment (40 large capsules a day) and nausea [13]. These attributes impact upon drug adherence which, in the case of UCDs, can be potentially fatal [14]. Similarly, NaBZ has been known to provoke gastritis, making it difficult for patients to orally tolerate the drug [3]. Given the reported problems with pre-existing drugs, health-related quality of life of patients and their carers could be significantly compromised. Research to date concerning health-related quality of life (HrQoL) in UCD patients has been sparse with research focussing on clinical outcomes such as ammonia levels and associated side-effects of medication. However, as survival rates have improved dramatically in people with UCD, there is a need to focus on improving HrQoL [11].

With regard to HrQoL, progress has been achieved in improving the tolerability of pharmacological interventions required to manage ammonia levels. Current pharmaceutical treatments for UCDs can be administered in a variety of formulations, with NaBZ and NaPBA requiring preparation prior to administration (see Table 1). Efforts to improve preparations so that they are more palatable for the patient have been made. For example, administering NaBZ as an oral solution and presenting patients with various flavours, as opposed to unflavoured, has been shown to improve acceptance [15]. Glycerol phenylbutyrate (GPB; marketed as Ravicti**®**) has demonstrated its safety and utility in controlling ammonia levels in UCD patients since first approved by the United States Food and Drug Administration (FDA) in 2013 [16, 17]. Marketing authorisation for GPB was issued by the European Medicines Agency (EMA) in 2015 [18]. GPB differs from both NaBZ and NaPBA in that it is taken in a liquid form via a syringe and can be taken at a much lower dosage and has minimal taste (Table 1).Preliminary research has demonstrated that treatment-related symptoms, such as nausea and abdominal pain, have decreased significantly once patients transitioned from NaPBA to GPB in both adults and children [19].

**Table 1:** Key drug features of sodium benzoate (NaBZ), sodium phenylbutyrate (NaPBA) and glycerol phenylbutyrate (GPB)

	Sodium benzoate^a^ NaBZ^b^	Sodium phenylbutyrate [20] NaPBA^b^	Glycerol phenylbutyrate (Ravicti®) [21] GPB^b^
European Medicines Agency (EMA) approval date	NA^a^	1999 [20]	2015 [21]
Dosage	250 mg/kg/day maximum: 12 g/day [3]	450–600 mg/kg/day in children weighing less than 20 kg 9.9–13.0 g/m^2^/day in children weighing more than 20 kg, adolescents and adults [20] The safety and efficacy of doses in excess of 20 g/day (40 tablets) has not been established [20]	The recommended total daily dose is based on body surface area^c^—ranges from: 4.5 ml/m^2^/day to 11.2 ml/m^2^/day [5.3 g/m^2^/day to 12.4 g/m^2^/day) [21]
Formulation/Preparation	Powder (dissolved in liquid and oral administration). Dispensed differently by for example hospital pharmacies^a^	Tablets or granules (measure with a dosing spoon and mix with solid foods or liquid foods) for oral administration [20]	Oral liquid (measure with a syringe) [21]
Frequency of administration	3–4 times per day [3]	With each meal or feeding (e.g., 4–6 times per day in small children) [20]	With each meal or feeding (e.g., 3 to 6 times per day) [21]
Taste	May taste salty, sweet, bitter, sour and sticky [22]	Bad taste [23]	Minimal taste [24]
Efficacy	Not evaluated in randomized clinical trials^a^	No randomized control trial was included for the EMA approval for NaPBA but the pivotal study for GPB met its predefined endpoint of non-inferiority to NaPBA with respect to ammonia control [21, 23]	Phase 2 comparison showed noninferiority of GPB to NaPBA with respect to ammonia control [21, 24] Post hoc analysis demonstrated ammonia was lower after treatment with GPB compared to NaPBA [21, 24]

^a^No Summary of Product Characteristics is available as NaBZ is not an approved drug. Available as special needs product in UK and may be available as named patient use in additional countries. NaBZ is a chemical and food preservative

^b^NaBZ and NaPBA are both solid and measured in grams (g). GPB is a liquid and measured in millilitres (ml)

^c^Body surface, square meter (m^2^) is used for GPB dosing. This measurement is used when the pharmacokinetic parameters increase in proportion with increasing body surface rather than weight

The burden of pharmacological treatment is typically high for UCD patients who must follow strict dosages and times, often coupled with a rigorously planned protein-restricted diet. A recent study that analysed data across international centres demonstrated that the burden of disease and the burden of dietary restrictions contributed significantly to impaired HrQoL [25]. The burden on both child and carer is such that many families consider the burden of disease a core deciding factor when considering whether their child should have a liver transplant rather than continue with medical management [26]. Research specifically into burden of pharmacological treatment has been sparse and thus requires further investigation in relation to HrQoL. As there are several treatment options available (NaPBA, NaBZ, GPB), capturing a breadth of experience utilising different drugs is pertinent. The psychosocial impact of pharmacological treatment has yet to be elucidated in the context of UCDs, where preliminary clinical evidence points toward elevated burden associated with particular treatments [13, 19].

HrQoL has been explored in UCD patients previously but primarily through survey studies rather than qualitative reports [27–29]. Interviews, as opposed to surveys, allow participants the freedom to express their experiences, within context, which may not have been previously considered [30]. The impact of pharmacological management on HrQoL in UCD patients who have transitioned from either NaPBA or NaBZ onto GPB has not been investigated. By gaining an understanding of how pharmacological management impacts HrQoL, practitioners will be better informed when deciding patient’s pharmacological treatment regime, considering the subsequent impact of the drug on HrQoL and which treatment approach would be best suited for each patient. Understanding the wider impact on other elements of HrQoL would also inform practitioners regarding the specific challenges patients face that may not have been previously considered beyond the immediate impact of drug intake, such as social and psychological implications. This study aims to explore the burden of pharmacological treatment and the effects of the transition to GPB on HrQoL in people with a UCD.

## Method

### Design

A qualitative design was selected using semi-structured interviews to address the research aim. Ethical approval was obtained from Manchester Metropolitan University Faculty Ethics Committee, UK (Ref: 10306). The study is reported in accordance with the consolidated criteria for reporting qualitative (COREQ) research [31].

### Participants

Participants were recruited utilising a purposive sample facilitated by Metabolic Support UK (MSUK). Participants were included if they, or someone they care for, had received a formal diagnosis of a UCD, they lived in the UK and had transitioned onto GPB as their primary form of treatment for the condition. As GPB is a relatively new treatment option, no time frame was set for a transition period. Participants were excluded if they were diagnosed with an inborn error of metabolism other than UCD or if they did not live in the UK. Given that it is estimated that UCD affects 2100 patients across Europe [32], the research team were aware that identifying and recruiting sufficient participants located in the UK would be challenging. A gatekeeper at MSUK, who was a respected member of the UCD community, was used to facilitate access to participants. A sample size of 10–15 participants was sought for the study, however recruitment continued until data saturation was achieved. Data saturation can be described as the point at which new information or data provokes little or no change to subsequent codes and themes [33]. The gatekeeper advertised the study in a private social media group (Facebook) that is well-established in the UK for carers of children with UCD. The advert stated that either carers or children with UCD were eligible to take part in the research. Recruitment for the study continued concurrently whilst interviews were being conducted, until data saturation was achieved. Members of the group were encouraged to get in contact with the gatekeeper if they were interested in participation once they had reviewed the brief advertisement. Emails containing expressions of interest were passed onto the research team who provided a participant information sheet and consent form to potential participants. Recorded audio informed consent was obtained over the phone prior to undertaking the interviews.

### Data collection

Semi-structured telephone interviews were arranged with participants and conducted by a single researcher from the team (DB). Telephone interviews were selected owing to the geographically dispersed nature of the sample. Telephone calls mimic the format of a semi-structured interview, whereby the caller adapts and reorders questions in alignment with the topic of discussion [34]. An interview guide was developed from a rapid scoping review of the UCD literature [11, 35, 36], coupled with expertise from the research team (see Appendix 1). Two pharmaceutical representatives who were experts in UCD pharmacological treatment were consulted regarding the interview guide to ensure its appropriateness. The interview guide was then reviewed by the gatekeeper from MSUK and a carer of a person living with UCD to ensure its suitability. The final interview guide was structured into three broad topics: living with UCD, HrQoL whilst utilising previous treatment(s) and, HrQoL whilst taking GPB. A semi-structured approach to the interview, afforded the researcher scope to explore elements of the participants’ lived experience that may not have been outlined in the interview guide. All interviews were digitally audio recorded and transcribed verbatim.

### Analysis

Thematic analysis was conducted on written transcripts to elucidate pertinent aspects of participant’s lived experience. The six phases of thematic analysis outlined by Braun and Clarke [37] were followed to facilitate methodological rigour. Written transcripts were read multiple times, immersing the researcher with the raw data prior to initial coding taking place. Codes were arranged into sub-themes and themes and reviewed by two researchers from the team (DB, GY), refining through discussion. Any conflicts were duly resolved with the help of the third member of the research team (FF). NVivo 12 software was used to organise transcripts, codes and themes accordingly. Analysis was performed iteratively throughout data collection as interviews were completed across a 3-month period owing to practical constraints, such as carers struggling for time during school holidays. This also allowed the research team to determine when data saturation was achieved.

## Results

Nine participants completed interviews across the months of June to August 2019 after expressing an interest in participation and indicating their consent to take part. Interviews were approximately 60 min long. The interview guide and coding framework were not modified during this time as no new topics were identified in the emerging data. All participants were carers for a child living with UCD. All children with UCD in this study were of infant age or had a learning disability and were not interviewed directly. However, some children were present for the interview and contributed where they wanted to. All children with UCD had transitioned from either NaPBA or NaBZ onto GPB. Demographic information relating to the UCD children can be found in Table 2. To protect participant’s anonymity, age range categories have been included for each participant to contextualise their experiences in relation to their stage of development (see Table 2). For clarity, participants are referred to as carers and the UCD patient that they care for are referred to as the child/children.

**Table 2:** Demographic data of child with UCD (all interviews undertaken with carers)

Age range categories^a^	Mean age	Sex	Previous treatment
P1: Adult P2: Primary school age P3: Primary school age P4: Primary school age P5: Primary school age P6: Pre-school age P7: High school age P8: Primary school age P9: Adult	12.44 (range 3–36, SD = 10.26)	*M* = 7 *F* = 2	*Sodium phenylbutyrate* (NaPBA) = 5 *Sodium benzoate* (NaBZ) = 4

^a^Pre-school age = 3–4 years old; Primary school age = 5–11 years old; High school age = 11–16 years old; Adult ≥ 16 years old

verbatim quotes from participants

Analysis of the data demonstrated that data saturation had been reached in order to answer the research question. As analysis was performed iteratively throughout the process, the research team kept a log of emerging themes to identify when saturation had been reached whilst interviews were being undertaken. Three themes were developed from the data: psychological health, physical health, and social participation (Table 3). Several subthemes were identified within each of these themes and have been used to present the findings along with anonymised.

**Table 3:** Themes and subthemes retrieved from interview data

Themes	Subthemes
Psychological health	Administration Emotions (worry, fear, loneliness and happiness)
Physical health	Overall condition Development
Social participation	School Family and friends Practicalities and planning

## Theme 1: Psychological health

### Administration

Managing UCD requires constant monitoring and high volumes of medication. Carers had historically relied upon either NaBZ or NaPBA to manage their child’s ammonia levels. However, carers described a multitude of negative components associated with these, ranging from high volume (“20mls was quite a large syringe” P2), a pungent smell (“it smelt absolutely terrible…” P9), unpalatable taste (“he used to complain that it burnt…” P8) and nausea (“after having it…he had tummy ache” P3). These factors converged in a way that rendered administration of the previous treatments highly challenging for the carer and distressing for the child. The challenge of giving the medication to their child was described by eight carers:

I just forced him, you had basically hold him down and force it into him, that’s was what you had to do even whenever we were in hospital that’s what we were just told “he has to take this” and we were having to hold him literally, which obviously caused a lot of distress for us and caused a lot of distress for him and distress around us, you know it’s just not a nice thing to have to do. (P6, pre-school age)

The psychological burden of restraint during administration was extremely high for the child, the carer administering the drug and onlookers such as family members. A number of carers resorted to spacing doses out to ensure that the medication was taken, by dripping smaller quantities across the day: “we used to literally just drip it in, because that was the only way we could get him to tolerate it”, P1. The specific administration times also presented a major inconvenience for carers:

it did require a very structured process of the set times and so, you know, there were days when at 9 o’clock at night all you want to do is go to bed but actually you can’t. Or you’ll end up going to bed and setting the alarm to get up at 11 o’clock to give [child’s name] his meds. (P2, primary school age)

The transition to GPB had alleviated the numerous challenges associated with administering previous treatments for many carers. GPB was reported to possess positive qualities in comparison to previous treatments including: better taste (“he said it tastes like strawberries…”, P8), reduced volume (“it’s a lot less, it’s just easy having a little syringe…”, P3) and reduced sickness (“I can knowingly say he’s took it [GPB] and [hasn’t] vomited [after taking it].”, P1). The perceived drastic improvement in medication led to less apprehension from children when taking their medication. Some carers also noted how GPB is received ready to administer, facilitating administration: “we haven’t got all that worry of making sure it’s made up to go anywhere” (P1). A recurring analogy used throughout by a few carers was the “battle” of administering medication on previous treatments, and noted how this battle had either been reduced greatly or eliminated altogether after the transition to GPB:

…we don’t have battles that we had sometimes three times a day. I can give it him on an empty stomach, you know, if we’re late with a dose or for some reason we haven’t got it next to us for some reason, which means we can be a bit more relaxed. It’s just a bit less stressful and less worrying. (P3, primary school age)

The rigorous schedule that carers had to follow when giving previous medications to ensure that ammonia levels were stable were not as rigorous when taking GPB. Given GPB’s slow release capabilities, carers felt more at ease giving medication slightly later or earlier: “…we now give [child’s name] his medication at 10 o’clock at night before I go to bed rather than my wife going to bed and I’m having to stay up another hour” (P2).

One negative aspect was described by a few carers when discussing sourcing specific syringes needed to administer GPB. As these syringes, which were designed to fit the bottle of GPB, were so difficult to find, it provoked stress for some carers as they were unable to administer medication as planned:

…the level of stress it created [obtaining the syringe] and does still continue to create when we get low because we’re almost going to [the prescribing hospital] to beg, borrow and steal a syringe …to be able to give [child’s name] his medication (P2, primary school age)

### Emotions—Worry, fear, loneliness & happiness

For carers and children living with UCD, the daily challenge of managing the condition and navigating a “normal” life led to substantial levels of worry. Especially following diagnosis and in the early stages of the condition, the uncertainty of how their child’s condition would fluctuate was perturbing for all carers. One carer described living their life on “a knife’s edge” and taking it “day to day” (P9). Worry was especially prominent concerning previous treatments for a number of carers, owing to side-effects and problems administering the old drug. Sickness associated with the old treatments also led to worry in carers:

“He instantly felt sick, and that was something that we used to worry a lot about, because it made the whole thing a lot worse…” (P3, primary school age)

Not only was nausea a worry in itself, carers also had anxiety surrounding whether they should re-administer medication after vomiting. When compared to the degree of worry experienced on GPB, some carers reported a burden being lifted given that administration was significantly less challenging:

The Ravicti [GPB] is fine, it’s a tiny amount, he can drink water after it’s gone. So yeah, that was a real weight off our mind that it was a great medicine really. (P8, primary school age)

Closely linked to the feeling of worry was the notion of fear, experienced by both the carer and external onlookers. UCDs are extremely rare metabolic disorders leading to both misunderstanding and a general lack of understanding from those who encountered it. The severity of the condition was especially concerning with a few carers reluctant to let their child be under another party’s temporary care as they felt that others did not truly comprehend the importance of monitoring their child’s condition:

I think (if it’s) outside the [family] unit, it frightens people. Cause you have to tell people what they might have to deal with… (P1, adult)

The condition also led to concerted feelings of fear from the carer themselves. Some carers stated feeling fearful of their child’s condition as they constantly anticipated possible complications. One carer described their apprehension over returning their child to bed after they had been unwell “…in case he would get sick” (P3). A clear link between worry and fear was observed, two negative emotions that impacted on life events. Important to note was the pervasive nature of these emotions which, some argued, would never change owing to the relentless battle with the condition as “that's just the UCD life I think.” (P5), irrespective of the medication that their child was taking. Caring for a child with UCD led to a couple of carers expressing their loneliness. The perceived lack of understanding from others contributed to a feeling of isolation for most carers: “It’s quite lonely. There’s nobody I can turn to that understands exactly what I’m going through” (P4).

Whilst negative emotions appeared to be an ever-present when managing a chronic life-threatening condition, positivity was expressed by a number of carers in relation to their transition to GPB. This was related to the burden of administration being reduced considerably leading to happier carers and ultimately, happier children:

He’s even happier now that he doesn’t have to stomach the Benzoate. Yeah, so you know, he’s happy. (P7, high school age)

It’s good for me not to see my son struggle taking the drug and that is really good to see. My quality of life is the same as before, but obviously not seeing your son struggle is brilliant. (P7, high school age)

## Theme 2: Physical health

### Overall condition

As UCDs are particularly volatile in nature, managing ammonia levels was a constant for the family unit. A few carers, with varying levels of success, described ammonia levels on previous treatments. For some, they felt that the UCD was managed appropriately with the previous treatments, whilst others reported slightly elevated ammonia levels:

It had been taken up to around the 80’s on the sodium benzoate but it was down to the 40’s and it has hovered around between 40 and 60 since, which is a good level. (P6, pre-school age)

A number of carers also noted how their child’s ammonia levels have reduced since the transition to GPB. They felt that the slow release properties of GPB controlled ammonia levels in a more stable manner: “since we’ve been on Ravicti [GPB] her ammonia levels have been the best they’ve ever been” (P4, primary school age).

### Development

The effect of elevated ammonia in the blood can be highly damaging for the patient leading to brain damage if left untreated. One carer specifically referenced how they felt that the NaBZ inflicted damage on their child and subsequently impacted their development:

I think for all the good that benzoate was doing when it was high in the body, when it was low, that damage was being done and that damage was being permanent… (P2, primary school age)

The same carer noted that, since their child had transitioned onto GPB, they felt their development had improved dramatically. This improvement had led to a substantial improvement in their quality of life, as the carer felt that their child would better comprehend experiences outside of the family home:

We’re taking him to the London Eye next week and then onto the aquarium for a day out because we understand, we feel that he will comprehend some of what he’s seeing. (P2, primary school age)

## Theme 3: Social participation

### School

Managing the condition had, for all carers, impacted upon school at one point or another. Children and carers experienced schooling-related issues related to pharmacological management such as: reduced attendance, managing medication, lack of support and difficulty attending extracurricular activities. For example, one carer described how their child’s attendance at school “stands at 56%” (P4), due to the complications experienced as a result of UCD and associated medication. Constant nausea and subsequent sickness after medication was the main contributing factor to non-attendance in this instance. Not only does sickness impact on attendance but “the number of [hospital] appointments” (P6) that children attended also reduced attendance at school.

In terms of the school’s involvement with managing the condition, carers had varying levels of success in communicating the importance of rigorous maintenance in terms of medication and diet. Problems had arisen regarding the school’s involvement in overseeing children’s strict dietary restraints and the administration of medication in a timely manner. One carer outlined how they felt the school simply did not understand the true nature of the condition and underestimated how serious a missed dose may be:

We’ve had instances of missed medication where I’ve been called to say “really sorry but we completely forgot to give him his medication and does he need to have it or can he have it when he gets home?” The seriousness of the condition has never really hit home. (P8, primary school age)

This carer also described feeling as if she were perceived as “neurotic” for monitoring her child’s diet so closely, especially when the school had considered giving the child food that other children were consuming at the time: “we used to have comments like “oh you need to loosen up a little bit; you’ll give him an eating disorder” (P8). The transition to GPB had alleviated stress in relation to administering the medication whilst the child was at school, as the midday dose which was required when taking NaPBA was no longer necessary. GPB also possesses slow-release qualities, as opposed to both NaBZ and NaPBA, meaning that the requirement to administer the medication at specific times throughout the day was no longer a worry:

…it’s a lot easier during the day to give him a simplified medication dose because the Ravicti [GPB] isn’t given until 4 o’clock, so yeah, I’d say it’s dramatically impacted on his life. (P8, primary school age)

This carer also remarked that the transition to GPB had enabled greater participation in extracurricular activities and school trips:“…if he has a school trip he’d have it 8 o’clock in the morning his next dose would be when he comes home at half three.” (P8) The carer noted that prior to the transition to GPB, they had to pack NaPBA with an ice pack to ensure it was stored at a temperature under 26 degrees, ready for administration.

### Family and friends

The management of the UCD was not limited to affecting the child. The ramifications of the condition placed an emotional toll on family members including carers and siblings of the child. One carer remarked how their daughter who’s sibling lived with UCD “had to start to cope with staying at friends’ houses at the last minute quite young… because we were in hospital for a few days” (P3). The battle to maintain positive emotion amongst multiple children, where the child with UCD must be monitored on a regular basis, was inevitably difficult.

The pressure that families felt to share tasks when looking after the child was also exceptionally high. Given the monitoring schedule to maintain their child’s health, carers shared the load to ease the burden on the family unit:

It might mean us having to, kind of, split our time, and I go away with [child’s name] and do stuff, dad goes away with [sister], and vice versa and then swap over. (P5, primary school age)

often we’d take it in turns we’d pass the imaginary baton… (P8, primary school age)

The sharing of the load was also fairly restricted to within the immediate family unit as tasks and practices required when looking after the child with UCD, such as the administration of highly specific doses of medication, were difficult for those unfamiliar with the condition. A number of carers recalled the issues they had encountered when trying to involve family with caring responsibilities.

I just had the whole burden really and we didn’t have a lot of local family to support us, and the ones that were local didn’t quite understand and never visited us in hospital or nothing… (P5, primary school age)

Evidence suggested that social participation with friends and family had become more likely since the transition to GPB. One account related to a carer’s attempts to take their child to the park to play. Owing to the child’s ongoing illness and negative experience with the previous treatment before GPB, this was almost impossible: “…whenever he got to the park he got straight off the trike and vomited, he was always very nauseous and very sick, he would have vomited every day” (P6). The carer described how, now that their child had transitioned onto GPB, trips to the park were possible as sickness had stopped:

He would maybe be out on his wee bike or whatever and he would just be sick. And as soon as he changed over to Ravicti [GPB] this sickness completely stopped. (P6, pre-school age)

In another account, the carer described how their child’s development since taking GPB had led to more quality time with their sibling. They were resolute that they would not go back to previous treatments given the benefits they had experienced since taking GPB:

[Child with UCD would] stand in the garden and scream “[sister’s name] come here” because he wants her to play in the garden with him. We are seeing massive changes and given a choice, would I go back [to the previous treatment]? Would I hell. You know [1] my only regret with Ravicti [GPB] is that [child’s name] didn’t have it 4 years ago. (P2, primary school age)

### Practicalities & planning

The treatment regime that children normally followed included: frequent medication, dietary restriction and hospital appointments. Balancing all of these aspects was challenging, and the practical facets of each presented novel challenges for carers involving detailed planning and preparation for even small trips outside of the home. This ranged from being extremely cautious when eating out (“we have to check in advance what’s on the menu”, P1) to packing additional items to make trips out of the home easier should the child become unwell (“I used to carry a spare sheet along in my change bag when she was born, because she would vomit that often”, P5). Two carers noted that holidays abroad were now practically impossible, owing to the range of considerations and paperwork they would have to complete to ensure a safe trip: “I don’t have the courage to go abroad because I don’t know what the medical…, where they are or how capable they are or getting the drugs through customs…” (P7). Ensuring that there was enough medication prepared for a trip out was also a barrier to engaging in activities outside of the home: “…going out’s a minefield you’re constantly checking have we got medication?” (P9). However, a few carers mentioned that, since the transition onto GPB and the decreased dosage, trips out of the house had become a lot easier:

So dramatically [improved child’s quality of life], because it’s taken away that midday dose, so for his medication to be simpler… it’s really simple what he has now during the day. (P8, primary school age)

Again, whilst GPB had contributed to less preparation (“it’s easier and you haven’t got all that making it up…”, P1), one carer noted that the planning and practicality aspect would persist irrespective of the treatment their child received:

It's just always going to be a constant, I think, I don't think any change in medication's going to help. I think it's just it's always going to be like that. (P5, primary school age)

## Discussion

Living with a UCD can be extremely debilitating depending on the severity of the condition. Intense pharmacological treatment, coupled with strict dietary control, means that both carers and children living with UCD are in a constant state of monitoring. Whilst HrQoL has previously been investigated primarily through survey studies, which can be limited in exploring experiences in-depth within context [27, 28, 30], this study successfully explored the burden of pharmacological treatment and subsequent impact on HrQoL in children living with UCD and their carers. After completing semi-structured interviews with carers of nine children living with UCD who had successfully transitioned from one medication to another, themes concerning psychological health, physical health and social participation emerged. Mainly positive aspects were described by carers in relation to the transition from either NaPBA or NaBZ to GPB when considering HrQoL. One negative element emerged regarding the specific syringes needed to administer GPB and experiencing difficulties acquiring them. This induced a level of stress in carers trying to manage their child’s ammonia levels. Children and carers were affected simultaneously in a variety of ways during pharmacological treatment and transition, demonstrating the burden placed on both the child and the individual administering medication, typically the carer.

The psychological impact of managing a UCD through pharmacological intervention, drawing from the lived experience of carers, was exceptionally high. Administration emerged as one of the main psychologically challenging aspects of pharmacological management. Linked implicitly to the negative attributes attributed to UCD treatments [19], adhering to a strict administration regime placed significant psychological strain on carers. One account described the act of having to physically restrain their child whilst taking NaBZ to ensure that the medication was taken as prescribed. For many, the most challenging aspect of pharmacological management was the administration of the drug. Previous research has illustrated how negative attributes of a drug can be significant barriers to adherence in patients with inborn errors of metabolism, such as the taste of the medication, the frequency of administration and associated side effects [14]. The problems associated with medication in the current study population (tolerability, dosage, nausea and smell), and the child’s general reluctance to take their medication, were often attributed to elements such as poor taste, burden of treatment and subsequent nausea experienced after the medication had been administered. The “battle” that many carers used metaphorically induced a deep sense of worry, whilst children experienced trepidation at administration times. It is important to note that the administration “battle” was alleviated somewhat after the transition onto GPB. In line with research describing the more positive elements of GPB [19], relying on GPB as primary pharmacological treatment could provide some respite for carers.

Negative emotions were frequently described and alluded to by carers, illustrating the multifaceted nature of the condition and pharmacological regime. Fear was an emotion both encountered and experienced by carers. Given the rigorous pharmacological management regime that is adhered to, many acquaintances, friends and family were scared to take full responsibility for the child living with UCD through fear of mismanaging the condition. The psychological and pragmatic burden of this was substantial, with carers shouldering the entirety of care responsibilities. Coupled with this were feelings of loneliness owing to the rarity of the condition and misunderstanding from others, as well as inordinate amounts of worry, especially when children experienced side effects that impacted upon subsequent administration times and doses.

The primary aim of pharmacological management in UCD is to maintain stable ammonia levels, thus preventing potential complications such as hyperammonemic episodes. The struggle to maintain safe ammonia levels was described in great detail by carers. Carers remarked how, on the initial treatment prior to the transition to GPB, ammonia levels were higher and would fluctuate more frequently. The impact of elevated ammonia levels in children living with UCD culminated into frequent hospitalisation and added burden for both child and carer. A number of participants noted that their child’s ammonia levels had reduced and become more stable following their transition onto GPB. This mirrors research suggesting that ammonia levels are significantly lower on GPB as compared to NaPBA, with reduced instances of abnormal values [38]. The consequential effect of improved ammonia levels for children living with UCD meant reduced hospital visits and alleviated worry for the carer. Again, it could be suggested that relying primarily on GPB as the main mode of pharmacological management reduces burden on both children and carers by reducing engagement with tertiary care and a reduction in negative emotions experienced, such as worry.

The effect of pharmacological management manifested itself in severe impacts on social participation including school, family events and holidays. Families described how difficult managing relationships with the child’s school was, as some schools failed to fully understand the severity of the condition. Whilst providing schools with a day-to-day management plan is recommended [3] and was commonly reported by carers, their experiences suggested that knowledge underpinning pharmacological treatment for UCD in teachers and school staff was missing. The rigorous nature of the day-to-day plan, above and beyond teacher’s daily routine, seemed to cause major inconvenience which ultimately impacted upon the child living with UCD and their wellbeing. Moreover, carers had experienced instances where their child was unable to participate in extracurricular school activities, or even mainstream education despite carers feeling that this would benefit their child socially. This mirrors previous research where parents of UCD patients possessed great concern over the social challenges their child faced [35]. The disparity between schools and carers is evident, suggesting that further work should seek to bridge the gap between education providers and the UCD community to better support children both in their education and in their social wellbeing.

Considering the impact of pharmacological management on carers involved in administering UCD medication, HrQoL should be a core consideration alongside reducing the child’s ammonia levels. These findings suggest that GPB reduces carergiver’s stress due to improved characteristics relating to taste, frequency and administration method. Further research is required to fully understand how the transition to GPB alleviates negative affect for the caregiver as well as the child living with UCD. The results of this work will facilitate future work that could provide both practical and emotional support to families living with UCD. Findings suggest that transitioning to GPB could alleviate some pragmatic challenges faced by families but, ultimately, that battling the condition will persist throughout the child’s life. It is vital that pharmaceutical companies and decision makers, including clinicians, ensure that enough syringes are produced and made readily available to reduce anxiety for carers. Greater attention should be placed on providing families and carers specifically with specialist resources to cope with the daily challenges that the condition presents, such as emotional support and respite. Medical professionals should consider GPB as the most appropriate option when considering carer’s HrQoL but also, based upon the carer perspective, the child’s experience of taking medication for their condition. Improved taste, timing and administration of GPB appears to contribute towards greater medication adherence. Consequently, this should help manage ammonia levels in a more consistent way, which is key to reducing the risk of hyperammonemic episodes, further developmental damage and the negative impact this may have on quality of life.

There are limitations to this study. Four participants were relatively early in their transition from previous treatments to GPB (within 6 months), meaning that they may not have had extensive experience in discussing long-term effects of GPB on HrQoL as opposed to previous treatments. Given GPB is a relatively new treatment option in terms of accessibility, determining the long-term impact of GPB on HrQoL may only be attainable in time when more UCD patients are engaged with GPB as their primary treatment option. Coupled with this, participants were primarily interviewed during the summer months during school holidays. One child had yet to begin formal schooling. Understanding the full range of schooling challenges is imperative to improving the apparent misunderstanding that carers’ encounter on a regular basis.

The sample of participants in this study cared for UCD children who were primarily of school age. Evidence suggests that mortality rates in neonatal patients is fairly high compared to healthy counterparts [39] suggesting that a significant number of UCD patients do not survive through their early years without strict pharmacological management. Whilst life expectancy has yet to be assessed in times where greater treatment options are available, future research should attempt to recruit older UCD patients to understand HrQoL in later stages of life and for those who may be living in a more independent fashion. Similarly, experiences of living with UCD could be different between the different types of metabolic deficiency, such as between ornithine transcarbamylase (OTC), carbamoyl phosphate synthetase 1 (CPS1) and argininosuccinate synthetase (ASS). Stratifying the severity and type of condition could elucidate any specific challenges that may exist between conditions and mode of drug intake. Further research could also investigate how stress-reducing interventions are received by carers of children with UCD and their impact on stress levels.

## Conclusion

This study has illustrated the multifaceted and persistent challenges carers face in relying on pharmacological treatment to manage UCDs in their children. Improved understanding of HrQoL in carers of children with UCD will facilitate decision-making processes for those in clinical practice to prescribe medication that pays due attention to the carer’s HrQoL. Outreach work should be performed with key locations, such as schools, to increase understanding of the condition and alleviate the considerable burden carers face on a daily basis.

## Data Availability

The datasets generated and/or analysed during the current study are not publicly available due to the data containing personally identifiable information, but are available from the corresponding author on reasonable request.

## Appendix 1: Interview guide

### General opening and background information about child and UCD

- Tell me a little bit about your condition/childs’s condition
- Tell me a little bit more about how you/your child were/was diagnosed with a UCD
- Birth or late onset (how long after birth)?
- Delayed diagnosis?
- Early symptoms?
- Event that triggered e.g. childbirth, infection, stress? *(only applicable for late onset)*
- Tell me about your/their day-to-day life and how the UCD impacts upon you/them e.g. restrictions, psychological burden, family, social life
- How has your quality of life changed?
- From your perspective, how has your child’s quality of life changed? *(if carer)*

### Experience of managing the disorder (before GPB)

- How have you managed your/their condition throughout your/their life?
- Dietary restrictions?
- What medications to decrease ammonia levels have you stopped taking when starting GPB?
- Tell me about your/their experience of using other drugs
- Treatment burden?
- Strengths/weaknesses of the drug?
  o Taste?
  p Administration/Amount of medication per dosing?
  q Other aspects related to the medication? E.g. storage/accessibility
- Did you take your medication as prescribed?/Was it easy to take the medications as instructed by your doctor?
  o If no, explore why/If missed doses as described, what were the reasons for the missed dose?
- What are your perceptions on how well your condition was managed with previous treatment?
- How would you describe your/their quality of life on previous treatments?

### Transitioning to GPB and subsequent use

- What were your perceptions of GPB prior to commencing treatment with it?
- For what reason were you/they switched to GPB?
- Who was involved in this decision?
- What reasons contributed to the decision to treat your/their condition with GPB rather than previous drugs?
- How did you/they find the transition period between previous treatments and GPB?
- How has GPB affected your/their day-to-day life?
- How does GPB compare/differ with other medications that decrease ammonia levels that you/they have previously used?
- Treatment burden?
- Taste?
- Administration/Amount of medication per dosing?
- Other aspects related to the medication?
- How easy is it to take the medication as instructed by your doctor?
  o If missed doses were described for previous treatments, have the number of occasions for missed doses decreased?
- What are your perceptions on how well your condition is managed with GPB?
- How has GPB affected your/their quality of life? e.g. restrictions, psychological burden, family, social life.

## Abbreviations

UCD: Urea cycle disorders
NaPBA: Sodium phenylbutyrate
GPB: Glycerol phenylbutyrate
HrQoL: Health-related quality of life
MSUK: Metabolic Support UK
OTC: Ornithine transcarbamylase
CPS1: Carbamoyl phosphate synthetase 1
ASS: Argininosuccinate synthetase
